# Spoken word recognition without a TRACE

**DOI:** 10.3389/fpsyg.2013.00563

**Published:** 2013-09-02

**Authors:** Thomas Hannagan, James S. Magnuson, Jonathan Grainger

**Affiliations:** ^1^Laboratoire de Psychologie Cognitive, CNRS/Aix-Marseille UniversityMarseille, France; ^2^Department of Psychology, University of ConnecticutStorrs, CT, USA; ^3^Haskins LaboratoriesNew Haven, CT, USA

**Keywords:** spoken word recognition, time-invariance, TRACE model, symmetry networks, string kernels

## Abstract

How do we map the rapid input of spoken language onto phonological and lexical representations over time? Attempts at psychologically-tractable computational models of spoken word recognition tend either to ignore time or to transform the temporal input into a spatial representation. TRACE, a connectionist model with broad and deep coverage of speech perception and spoken word recognition phenomena, takes the latter approach, using exclusively time-specific units at every level of representation. TRACE reduplicates featural, phonemic, and lexical inputs at every time step in a large memory trace, with rich interconnections (excitatory forward and backward connections between levels and inhibitory links within levels). As the length of the memory trace is increased, or as the phoneme and lexical inventory of the model is increased to a realistic size, this reduplication of time- (temporal position) specific units leads to a dramatic proliferation of units and connections, begging the question of whether a more efficient approach is possible. Our starting point is the observation that models of visual object recognition—including visual word recognition—have grappled with the problem of spatial invariance, and arrived at solutions other than a fully-reduplicative strategy like that of TRACE. This inspires a new model of spoken word recognition that combines time-specific phoneme representations similar to those in TRACE with higher-level representations based on string kernels: temporally independent (time invariant) diphone and lexical units. This reduces the number of necessary units and connections by several orders of magnitude relative to TRACE. Critically, we compare the new model to TRACE on a set of key phenomena, demonstrating that the new model inherits much of the behavior of TRACE and that the drastic computational savings do not come at the cost of explanatory power.

## 1. Introduction

There is a computational model of spoken word recognition whose explanatory power goes far beyond that of all known alternatives, accounting for a wide variety of data from long-used button-press tasks like lexical decision (McClelland and Elman, 1986) as well as fine-grained timecourse data from the visual world paradigm (Allopenna et al., 1998; Dahan et al., 2001a,b; see Strauss et al., 2007, for a review). This is particularly surprising given that we are not talking about a recent model. Indeed, the model we are talking about—the TRACE model (McClelland and Elman, 1986)—was developed nearly 30 years ago, but successfully simulates a broad range of fine-grained phenomena observed using experimental techniques that only began to be used to study spoken word recognition more than a decade after the model was introduced.

**Figure 1:**
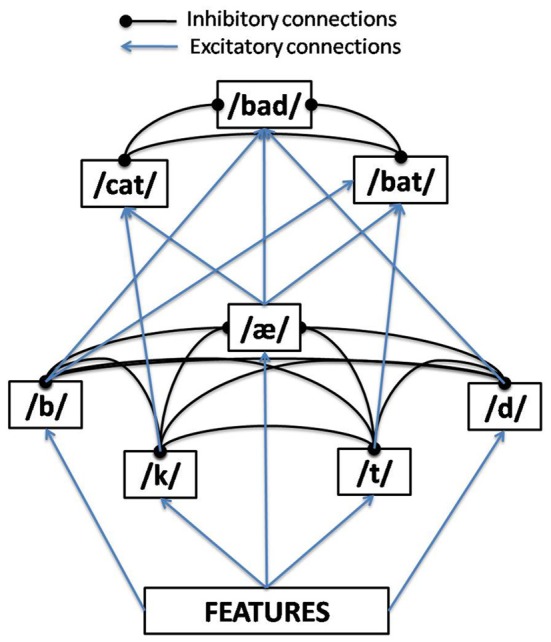
**One time-slice of the TRACE model of spoken word recognition**.

TRACE is an interactive activation (IA) connectionist model. The essence of IA is to construe word recognition as a hierarchical competition process taking place over time, where excitatory connections between levels and inhibitory connections within levels result in a self-organizing resonance process where the system fluxes between dominance by one unit or another (as a function of bottom–up and top–down support) over time at each level. The levels in TRACE begin with a pseudo-spectral representation of acoustic-phonetic features. These feed forward to a phoneme level, which in turn feeds forward to a word level. The model is interactive in that higher levels send feedback to lower levels (though in standard parameter settings, only feedback from words to phonemes is non-zero). Figure 1 provides a conceptual schematic of these basic layers and connectivities, although the implementational details are much more complex.

**Figure 2:**
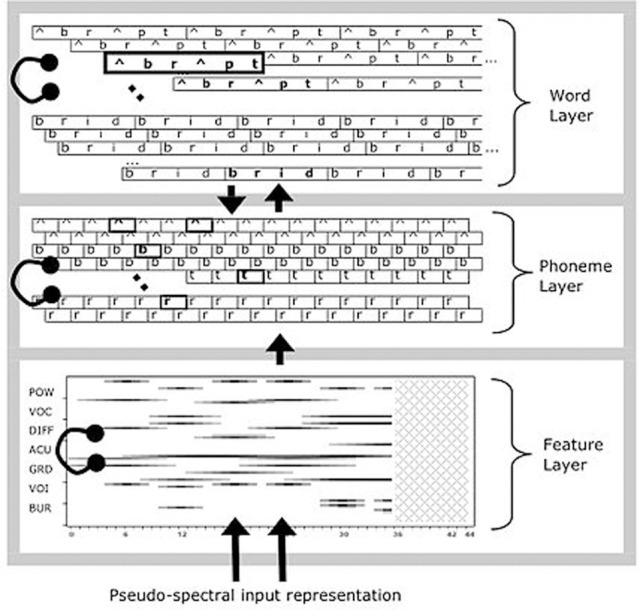
**The detailed structure of the TRACE model of spoken word recognition (adapted from McClelland and Elman, 1986)**.

The details are more complex because of the way the model tackles the extremely difficult problem of recognizing series of phonemes or words that unfold over time, at a sub-phonemic grain. The solution implemented in TRACE is to take the conceptual network of Figure 1 and reduplicate every feature, phoneme, and word at successive timesteps. Time steps are meant to approximate 10 ms, and feature units are duplicated at every slice, while phonemes and words are duplicated every third slice. Thus, the phoneme layer can be visualized as a matrix with one row per phoneme and one column per time slice (i.e., a phonemes × slices matrix). However, units also have temporal extent—features for a given phoneme input extend over 11 time slices, ramping on and off in intensity. The same scheme is used at the lexical level, which can be visualized as a words × time slices matrix. Word lengths are not the simple product of constituent phoneme durations because phoneme centers are spaced six slices apart. This also gives TRACE a coarse analog to coarticulation; the features for successive phonemes overlap in time (but this is a weak analog, since feature patterns simply overlap and sometimes sum; but real coarticulation actually changes the realization of nearby and sometimes distant articulatory gestures). Each feature unit has forward connections to all phoneme units containing that feature that are aligned with it in time. Each phoneme unit has a forward connection to and a feedback connection from each word unit that “expects” that phoneme at that temporal location (so a /d/ unit at slice *s* has connections to /d/-initial words aligned near [at or just before or after] slice *s*, /d/-final words whose offsets are aligned at or adjacent to *s*, etc.). This more complex structure is shown in Figure 2.

The input to the model is transient; activation is applied to feature units “left-to-right” in time, as an analog of real speech input. Features that are activated then send activation forward. In IA networks, activation persists even after the removal of bottom–up input, as activation decays gradually rather than instantaneously. So as time progresses beyond the moment aligned with slice *s*, units aligned at slice *s* can continue to be active. A unit's activation at a time step, *t*, is a weighted sum of its bottom–up input, its top–down input, and its own activation at time *t-1*, minus a decay constant. The crucial point in understanding TRACE is that time is represented in two different ways. First, stimulus time unfolds step-by-step, with bottom–up inputs for that step applied only in that step. Second, time-specific units at each level are aligned with a specific time step, *t*, but their activation can continue to wax and wane after the bottom–up stimulus has been applied at time *t*. This is because the model will only receive external input at time *t*, but activation will continue to flow among units aligned with time *t* as a function of bottom–up, top–down, and lateral connections within the model. This is what inspires the name “TRACE”: activation of a unit at time *t* is a constantly updating memory of what happened at time *t* modulated by lateral and top–down input.

In the original TRACE paper, McClelland and Elman presented results demonstrating how TRACE accounts for about 15 (depending on how one counts) crucial phenomena in human speech perception and spoken word recognition (see also Strauss et al., 2007 for a review). McClelland (1991) demonstrated how the addition of stochastic noise allowed TRACE to account properly for joint effects of context and stimulus (in response to a critique by Massaro, 1989). More recently, TRACE has been successfully applied to the fine-grained time-course of effects of phonological competition (Allopenna et al., 1998), word frequency (Dahan et al., 2001a), and subcateogorical (subphonemic) mismatches (Dahan et al., 2001b), using the visual world paradigm (Tanenhaus et al., 1995). In this paradigm, eye movements are tracked as participants follow spoken instructions to interact with real or computer-displayed arrays of objects (see Cooper, 1974, for an earlier, passive-task variant of the paradigm, the potential of which was not recognized at the time). While participants make only a few saccades per trial, by averaging over many trials, one can estimate the fine-grained time course of lexical activation and competition over time.

While some models have simulated aspects of visual world results (e.g., ShortlistB, Norris and McQueen, 2008), none has simulated the full set TRACE simulates, nor with comparable precision (although this assertion is based largely on absence of evidence—most models have not been applied to the full range of phenomena TRACE has; see Magnuson et al., 2012, for a review). While TRACE is not a learning model, its ability to account for such a variety of findings in a framework that allows one to test highly specific hypotheses about the general organization of spoken word recognition (for instance TRACE's assumption of localist and separated levels of representations makes it easier to consider the impact of perturbing specific levels of organization, i.e., sublexical or lexical). However, while TRACE does an excellent job at fitting many phenomena, its translation of time to space via its time-specific reduplications of featural, phonemic and lexical units is notably inefficient (indeed, McClelland and Elman, 1986 noted it themselves; p. 77). In fact, as we shall describe in detail below, extending TRACE to a realistic phoneme inventory (40 instead of 14) and a realistic lexicon size (20,000 instead of 212 words) would require approximately 4 million units and 80 billion connections. To us, this begs a simple question: is it possible to create a model that preserves the many useful aspects of TRACE's behavior and simplicity while avoiding the apparent inefficiency of reduplication of time-specific units at every level of the model? As we explain next, we take our inspiration from solutions proposed for achieving spatial invariance in visual word recognition in order to tackle the problem of temporal invariance in spoken word recognition.

### 1.1. Time and trace: man bites god

Visual words have several advantages over spoken words as objects of perception. All their elements appear simultaneously, and they (normally) persist in time, allowing the perceiver to take as much time as she needs, even reinspecting a word when needed. In a series of words, spaces indicate word boundaries, making the idea of one-at-a-time word processing (rather than letter-by-letter sequential processing) possible. In speech, the components of words cannot occur simultaneously (with the exception of single-vowel words like “a”). Instead, the phonological forms of words must be recovered from the acoustic outcomes of a series of rapidly performed and overlapping (coarticulated) gymnastic feats of vocal articulators. A spoken word's parts are transient, and cannot be reinspected except if they are held in quickly decaying echoic memory. In a series of words, articulation and the signal are continuous; there are no robust cues to word boundaries, meaning the perceiver must somehow simultaneously segment and recognize spoken words on the fly. Any processing model of spoken word recognition will need some way to code the temporal order of phonemes and words in the speech stream. There are four fundamental problems the model will have to grapple with.

First, there is the “temporal order problem,” which we might call the “*dog or god*” problem. If, for example, a model simply sent activation to word representations whenever any of their constituent phonemes were encountered without any concern for order, the sequences /dag/, /gad/, /agd/ (etc.) would equally and simultaneously activate representations of both *dog* and *god*. TRACE solves this by having temporal order built into lexical level units: a unit for *dog* is a template detector for the ordered pattern /d/-/a/-/g/, whereas a *god* unit is a template detector for /g/-/a/-/d/.

Second, there is the “multi-token independence problem,” or what we might call the “*do/dude*” or “*dog eats dog*” problem: the need to encode multiple instances of the same phoneme (as in words like *dude*, *dad*, *bib*, *gig*, *dread*, or *Mississippi*) or word (as in *dog eats dog*). That is, a model must be able to treat the two instances of /d/ in *dude* and the two instances of *dog* in *dog eats dog* as independent events. For example, if we tried having a simple model with just one unit representing /d/, the second /d/ in *dude* would just give us more evidence for /d/ (that is, more evidence for *do*), not evidence of a new event. The same would be true for *dog eats dog*; a single *dog* unit would just get more activated by the second instance without some way of treating the two tokens as independent events. TRACE achieves multi-token independence by brute force: it has literally independent detectors aligned at different time slices. If the first /d/ is centered at slice 6, the /a/ (both /a/ and /ae/ are represented by /a/ in TRACE) will be centered at slice 12 and the final /d/ will be centered at slice 18. The two /d/ events will activate completely different /d/ phoneme units. Thus, TRACE achieves multi-token independence (the ability to “recognize” two temporally distant tokens of the same type as independent) by having time-specific detectors.

Third is the “*man bites dog*” problem, which is the temporal order problem extended to multi-word sequences. The model must have some way to code the ordering of words; knowing that the words *dog*, *man*, and *bites* have occurred is insufficient; the model must be able to tell *man bites dog* from *dog bites man*. Putting these first three problems together, we might call them the “*man bites god*” problem—without order, lexical ambiguities will lead to later phrasal ambiguities. TRACE's reduplicated units allow it to handle all three.

Finally, there is the “segmentation problem.” Even if we ignore the primary segmentation problem in real speech (the fact that phonemes overlap due to coarticulation) and make the common simplifying assumption that the input to spoken word recognition is a series of already-recognized phonemes, we need a way to segment words. It may seem that this problem should be logically prior to the “*man bites dog*” problem, but many theories and models of spoken word recognition propose that segmentation emerges from mechanisms that map phonemes to words. For example, in the Cohort model (Marslen-Wilson and Tyler, 1980), speech input in the form of phoneme sequences is mapped onto lexical representations (ordered phonological forms) phoneme-by-phoneme. When a sequence cannot continue to be mapped onto a single word, a word boundary is postulated (e.g., given *the dog*, a boundary would be postulated at /d/ because it could not be appended to the previous sequence and still form a word). TRACE was inspired largely by the Cohort model, but rather than explicitly seeking and representing word boundaries, segmentation is emergent: lateral inhibition among temporally-overlapping word units forces the model to settle on a series of transient, temporary “winners”—word units that dominate at different time slices in the “trace.”

Solving several problems at once is compelling, but the computational cost is high. Specifically, because TRACE relies on reduplication at every time slice of features, phonemes, and words, the number of units in the model will grow linearly as a function of the number of time slices, features, phonemes, and words. But because units in TRACE have inhibitory links to all overlapping units at the same level, the number of connections grows quadratically as units at any level increase. Scaling up the 14 phonemes in the original TRACE model to the approximately 40 phonemes in the English inventory would not in itself lead to an explosive increase in units or connections (see Appendix A). Moving from the original TRACE lexicon of just 212 words to a realistically-sized lexicon of 20,000 words, however, would. In fact, the original TRACE model, with 14 phonemes and 212 words would require 15,000 units and 45 million connections. Increasing the phoneme inventory would change the number of units to approximately 17,000 and the number of connections to 45.4 million. Increasing the lexicon to 20,000 words would result in 1.3 million units and 400 billion connections. How might we construct a more efficient model?

### 1.2. Visual and spoken word recognition

There are several reasons to believe that visual and spoken word recognition could share more mechanisms than is usually appreciated. To be sure, very salient differences exist between the visual and auditory modalities. One signal has a temporal dimension, the other is spatially extended. The former travels sequentially (over time) through the cochlear nerve, the latter in parallel through the optic nerve. In addition, just as in spoken word recognition, researchers in the field of visual word recognition have to ponder an invariance problem. Although a unique fixation near the center of a word is usually enough for an adult to recognize it (Starr and Rayner, 2001), ultimately this fixation has only stochastic precision and will rarely bring the same stimulus twice at exactly the same place on the retina, resulting in dissimilar retinal patterns. A credible model of the visual word recognition system should find a way to overcome this disparity in a word's many location exemplars, and to summon a unique lexical meaning and a unique phonology independently of wherever the visual stimulus actually fell on the retina.

### 1.3. String kernels

In the machine learning literature, one computational technique that has been very successful at comparing sequences of symbols independently of their position goes under the name of string kernels (Hofmann et al., 2007). Symbols could be amino-acids, nucleotides, or letters in a webpage: in every case the gist of string kernels is to represent strings (such as “TIME”) as points in a high-dimensional space of symbol combinations (for instance as a vector where each component stands for a combination of two symbols, and only the components for “TI,” “TM,” “TE,” “IM,” “IE,” “ME” would be non-zero). It is known that this space is propitious to linear pattern separations and yet can capture the (domain-dependent) similarities between them. String kernels have also been very successful due to their computability: it is not always necessary to explicitly represent the structures in the space of symbol combinations in order to compute their similarity (the so-called “kernel trick,” which we will not use here).

It has been argued that string kernels provide a very good fit to several robust masked priming effects in visual word recognition, such as for instance letter transposition effects (the phenomenon that a letter transposition like *trasnpose* better primes the original word than a stimulus with letter replacements, such as *tracmpose*), and are thus likely involved at least in the early stages of visual word encoding (Hannagan and Grainger, 2012). To our knowledge, however, there have been no published investigations of string kernels in the domain of spoken word recognition. While the notion of an open biphone may at first blush sound implausible, keep in mind that the open bigram string kernel approach affords spatial invariance for visual word recognition. Might it also provide a basis for temporal invariance for spoken words?

## 2. Tisk, the time invariant string kernel model of spoken word recognition: materials and methods

### 2.1. General architecture and dynamics

**Figure 3:**
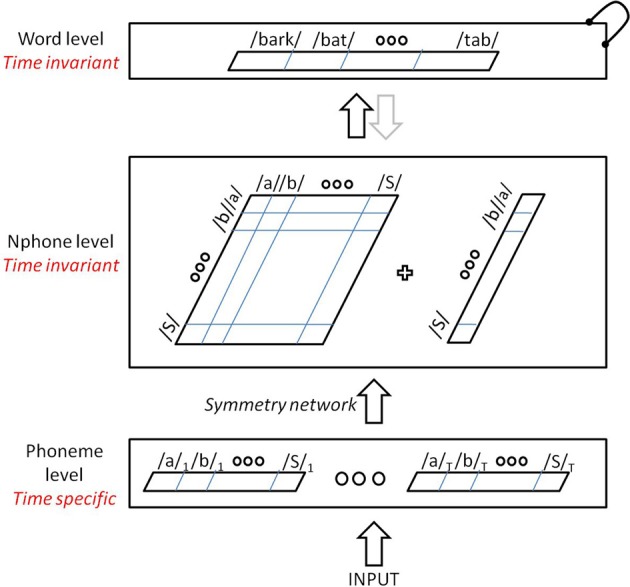
**The TISK model—a time-invariant architecture for spoken word recognition**.

Our extension of the string kernel approach to spoken words is illustrated in Figure 3. It uses the same lexicon and basic activation dynamics as the TRACE model, but avoids a massive reduplication of units, as it replaces most time-specific units from TRACE with time-invariant units. It is comprised of four levels: inputs, phonemes, nphones (single phones and diphones) and words. Inputs consist of a bank of time-specific input units as in TRACE, through which a wave of transient activation travels. However, this input layer is deliberately very simplified compared to its TRACE analog. The input is like the Dandurand et al. (2010) input layer, though in our case, it is a time slice × phoneme matrix rather than a spatial slot × letter matrix. Thus, for this initial assay with the model, we are deferring an implementation like TRACE's pseudo-spectral featural level and the details it affords (such as TRACE's rough analog to coarticulation, where feature patterns are extended over time and overlap). With our localist phoneme inputs, at any time there is always at most one input unit active—inputs do not overlap in time, and do not code for phonetic similarity (that is, the inputs are orthogonal localist nodes). Note that the use of time-specific nodes at this level is a matter of computational convenience without theoretical commitment or consequence; these nodes provide a computationally expedient way to pass sequences of phonemic inputs to the model, and could conceivably be replaced by a single bank of input nodes (but this would require other additions to the model to allow inputs to be “scheduled” over time). As in the TRACE model, one can construe these input nodes as roughly analogous to echoic memory or a phonological buffer. As we shall see, these simplifications do not prevent the model from behaving remarkably similarly to TRACE.

For our initial simulations, the model is restricted to ten slices (the minimum number needed for single-word recognition given the original TRACE lexicon), each with 14 time-specific phoneme units (one for each of the 14 TRACE phonemes). The input phoneme units feed up to an nphone level with one unit for every phoneme and for every ordered pairing of phonemes. The nphone units are time-invariant; there is only one /d/ unit at that level and only one /da/ diphone unit. Finally, nphone units feed forward to time-invariant (one-per-word) lexical units.

A critical step in the model is the transition between the time-specific phoneme input level and the time-invariant nphone level. This is achieved via entirely feedforward connections, the weights of which are set following certain symmetries that we will describe shortly. The nphone level implements a string kernel and consists of 196 + 14 units, one for each possible diphone and phoneme given the TRACE inventory of 14 phonemes. Units at this level can compete with one another via lateral inhibition, and send activation forward to the time invariant word level through excitatory connections, whose weights were normalized by the number of nphones of the destination word. The word level consists of 212 units (the original TRACE lexicon), with lateral inhibitory connections only between those words that share at least one phoneme at the level below. For this preliminary investigation, feedback connections from words to nphones were not included.

Units in the model are leaky integrators: at each cycle *t*, the activation *A*_*i*_ of unit *i* will depend on the net input it receives and on its previous activation, scaled down by a decay term, as described in Equation (1):

(1) $$A_{i}(t)=\{\begin{array}{ll} A_{i}(t-1)*(1-\text{Decay}) & \\ \;+\;{\text{Net}}_{i}(t)*(1-A_{i}(t-1)), & {\text{if Net}}_{i}>0\\ A_{i}(t-1)*(1-\text{Decay}) & \\ \;+\;{\text{Net}}_{i}(t)*A_{i}(t-1), & {\text{if Net}}_{i}\leq 0 \end{array}$$

where the net input of unit *i* at time *t* is given by:

(2) $${\text{Net}}_{i}=\sum_{j=1}^{k}w_{ij}A_{j}(t)$$

Python code for the model is available upon request to the first author, and a list of parameters is provided below as supplemental data. In the next section, we describe in detail the connections between time-specific phonemes and time-invariant nphones.

### 2.2. From time-specific to time-invariant units: A symmetry network for phonological string kernels

We now describe the transition phase between time-specific phonemes and time-invariant nphones in the TISK model. It is clear that unconstrained (that is, unordered) “open diphone” connectivity would be problematic for spoken words; for example, if *dog* and *god* activated exactly the same diphones (/da/, /dg/, /ag/, /ga/, /gd/, /ad/), the system would be unable to tell the two words apart. The challenge is to activate the correct diphone /da/, but not /ad/, upon presentation of a sequence of phonemes like [/*d*/_*t*_, /*a*/_*t* + 1_], that is, phoneme /d/ at time *t* and phoneme /a/ subsequently. Thus, the goal is to preserve activation of non-adjacent phonemes as in an open diphone scheme (for reasons explained below) with the constraint that only observed diphone sequences are activated—that is, *dog* should still activate a /dg/ diphone (as well as /da/ and /ag/) because those phonemes have been encountered in that sequence, but not /gd/, while *god* should activate /gd/ but not /dg/. This would provide a basis for differentiating words based on sequential ordering without using time-specific units “all the way up” through the hierarchy of the model.

The issue of selectivity (here, between “anadromes”: diphones with the same phonemes in different order) vs. invariance (here, to position-in-time) has long been identified in the fields of visual recognition and computer vision, and has recently received attention in a series of articles investigating invariant visual word recognition (Dandurand et al., 2010, 2013; Hannagan et al., 2011).

Directly relevant to this article, Dandurand et al. (2013) trained a simple perceptron network (that is, an input layer directly connected to an output layer, with weights trained using the delta rule) to map location-specific strings of letters to location-invariant words. To their surprise, not only did this simplistic setup succeed in recognizing more than 5000 words, a fair fraction of which were anagrams, it also produced strong transposition effects. By introducing spatial variability—the “i” in *science* could occur in many different absolute positions rather than just one—tolerance for slight misordering in relative position emerged. When Dandurand et al. (2013) investigated how the network could possibly succeed on this task in the absence of hidden unit representations, they observed that during the course of learning, the “Delta learning rule” had found an elegant and effective way to keep track of letter order by correlating connection strengths with the location of the unit. More precisely, the connections coming from all “e” units and arriving at word *live* had their weights increasing with the position, whereas the connections from the same units to the word *evil* had their weights decreasing with position. In this way, connection weights became a proxy for the likelihood of a word given all letters at all positions. This simple scheme enabled the network to distinguish between anagrams like *evil* and *live*. We describe next how this solution found by the delta rule can be adapted to map time-specific phonemes to time-invariant diphones or single phonemes.

**Figure 4:**
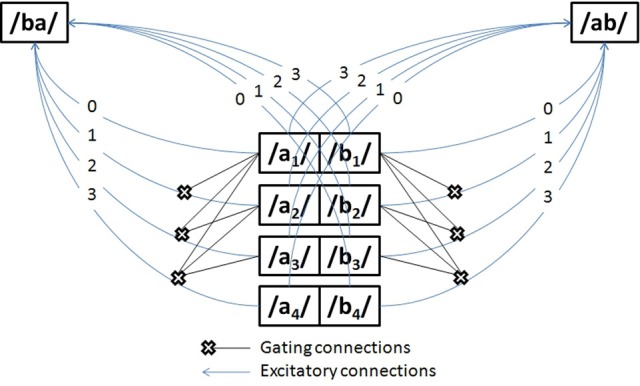
**A symmetry network for time-invariant nphone recognition that can distinguish anadromes.** The units in the center of the diagram (e.g., /*a*/_1_) represent time-specific input nodes for phonemes /*a*/ and /*b*/ at time steps 1–4. The /*ba*/ and /*ab*/ nodes represent time-invariant diphone units.

The network in Figure 4 has two symmetries: firstly, weights are invariant to changes in input phoneme *identity* at *any given time*. This is manifest in Figure 4 by the symmetry along the medial vertical axis: for any *t*, *a*_*t*_ and *b*_*t*_ can exchange their weights. Secondly, weights are invariant to changes in input phonemes *identity* across *opposite times* (in Figure 4), a central symmetry with center midway through the banks of input phonemes: for any *t* ≤ *T*, *a*_*t*_ and *b*_*T* − *t*_ are identical, and so are *b*_*t*_ and *a*_*T* − *t*_. Although the first symmetry concerns both excitatory (arrows) and gating connections (crosses, which will be shortly explained), the second symmetry concerns only excitatory connections.

What is the point of these symmetries? Consider a network where the weights have been set up as in Figure 4. Then at all possible times *t*, presenting the input sequence [/*a*/_*t*_, /*b*/_*t* + 1_] by clamping the appropriate units to 1 will always result in a constant net input for /*ab*/, here a net input of 4, and it will always result in a smaller constant net input to /*ba*/, here a net input of 2. A common activation threshold for every diphone unit can then be set anywhere between these two net inputs (for instance, a threshold of 3), that will ensure that upon perceiving the sequence [/*a*/_*t*_, /*b*/_*t* + 1_] the network will always recognize /*ab*/ and not /*ba*/. The same trick applies for the complementary input sequence [/*b*/_*t*_, /*a*/_*t* + 1_], by setting the weights from these phoneme units to the transposed diphone /*ba*/ in exactly the opposite pattern. A subtlety, however, is that in order to prevent sequences with repeated phonemes like [/*b*/_1_, /*a*/_2_, /*b*/_3_] from activating large sets of irrelevant nphones like /*br*/ or /*bi*/, it is necessary to introduce gating connections (cross-ended connections in Figure 4), whereby upon being activated, unit /*b*/_1_ will disable the connection between all future /*b*/_*t*_ >1 and all diphones /^*^b/ (where “*” stands for any phoneme but *b*).

The use of gating connections is costly, as the number of connections needed is proportional to the square of the number of time slices, but less naïve gating mechanisms exist with explicit gating units that would be functionally equivalent at a much smaller cost (linear with increasing numbers of time slices). More generally, other mappings between time-specific phonemes and time-invariant n-phones are possible. However, our approach is cast within the theory of symmetry networks (Shawe-Taylor, 1993), which ensures that several mathematical tools are available to carry out further analysis. The particular symmetry network introduced here arguably also has a head-start in learnability, given that it builds on a solution found by the delta rule. Specifically, in a perceptron trained to recognize visual words (Dandurand et al., 2013), the Delta rule found the “central symmetry through time” visible in Figure 4. We do not know if pressure to represent temporal sequences would allow the model to discover the “axial” symmetry and necessity for gating connections, but this is a question we reserve for future research. We note that some studies have reported the emergence of symmetry networks in more general settings than the delta rule and word recognition, that is, under unsupervised learning algorithms and generic visual inputs (Webber, 2000). Perhaps the best argument for this architecture is that it is reliable, and allows for the activation of the kind of “string kernels” recently described by Hannagan and Grainger (2012), at a computational cost that can be regarded as an upper-bound and yet is not prohibitive.

## 3. Results

### 3.1. Performance on single word recognition

We begin with a comparison of TISK and TRACE in terms of the recognition time of each word in the original 212-word TRACE lexicon. If TISK performs like TRACE, there should be a robust correlation between the recognition time for any particular word in the two models. We operationalized spoken word recognition in three different ways: an absolute activation threshold (*R*_abs_), a relative activation threshold (*R*_rel_) and a time-dependent criterion (*R*_tim_). The first criterion states that a word is recognized if its activation reaches an absolute threshold, common to all words. In the second criterion, recognition is granted whenever a word's activation exceeds that of all other words by a threshold (0.05 in the simulations). Finally the time-dependent criterion defines recognition as a word's activation exceeding that of all other words for a certain number of cycles (10 cycles in the simulations).

**Figure 5:**
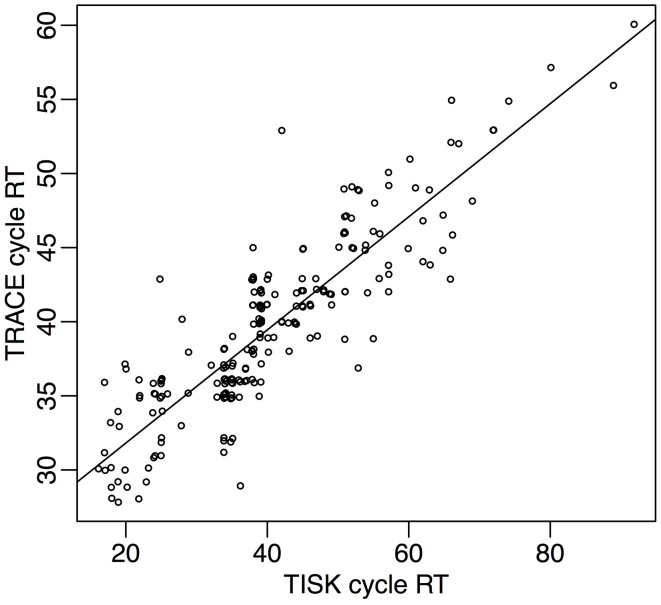
**Response times in TISK (*x*-axis) and TRACE (*y*-axis) for all 212 words in the lexicon, when a time threshold is used for recognition**.

Spoken word recognition accuracy for TRACE is consistently greater than that for TISK in these simulations, although both models obtain high performance under all criteria. TRACE exhibits close to perfect recognition with the three criteria (*T*_abs_ = 97%, *T*_rel_ = 99%, *T*_tim_ = 99%). TISK on the other hand operates less well under an absolute criterion, but recognition is improved using a relative threshold, and it rises to TRACE-like level with a time-dependent threshold (*T*_abs_ = 88%, *T*_rel_ = 95%, *T*_tim_ = 98%). Also, mean recognition cycles are similar for TRACE (*T*_abs_ = 38 cycles, *T*_rel_ = 32 cycles, *T*_tim_ = 40 cycles) and for TISK (*T*_abs_ = 45 cycles, *T*_rel_ = 38 cycles, *T*_tim_ = 40 cycles). At the level of individual items, performance is very similar for the two models, as revealed by high correlations between recognition times (for correctly recognized items) under all three recognition definitions (r for each definition: *T*_abs_ = 0.68, *T*_rel_ = 0.83, *T*_tim_ = 0.88). Figure 5 illustrates the correlation between response times in the case of *T*_tim_. In the rest of this article we will use the time-dependent criterion *T*_tim_, as the one with which models achieved both the best performance and the most similar performance.

It is also instructive to consider the two words on which TISK failed, /triti/ (*treaty*) and /st^did/ (*studied*). Indeed the model confused these words with their respective embedded cohort competitors /trit/ (*treat*) and /st^di/ (*study*). For the model these are the most confusable pairs of words in the lexicon, because in each case almost exactly the same set of nphones is activated for the target and the cohort competitor, except for one or two n-phones (the only additional diphone for *treaty* compared to *treat* is /*ii*/; *studied* activates two additional diphones compared to *study*: /dd/ and /id/). It is certainly possible to fine-tune TISK so as to overcome this issue. Note also that TISK recognizes correctly the vast majority of words containing embeddings, including word-onset embeddings.

But these particular failures are perhaps more valuable in that they point to the type of learning algorithm that could be used in the future, in TISK as in TRACE, to find the connection weights in a more principled manner. Namely, they strongly suggest that a learning algorithm should attribute more weight to these connections that are the most diagnostic given the lexicon (e.g., connection /ii/ to /triti/).

### 3.2. Time course of lexical competitors

As previously observed, what is impressive about the TRACE model is less its ability to recognize 212 English words than the way it does so, which captures and explains very detailed aspects of lexical competition in human spoken word recognition. Consider the so-called “Visual World Paradigm” (Tanenhaus et al., 1995), in which subjects' eye movements are tracked as they follow verbal instructions to manipulate items in a visual display. When the items include objects with similar sounding names (e.g., so-called “cohort” items with the same word onset, such as *beaker* and *beetle*, or rhymes, such as *beaker* and *speaker*) as well as unrelated items to provide a baseline, eye movements provide an estimate of activation of concepts in memory over time. That is, the proportion of fixations to each item over time maps directly onto phonetic similarity, with early rises in fixation proportions to targets and cohorts and later, lower fixation proportions to rhymes (that are still fixated robustly more than unrelated items; Allopenna et al., 1998). Allopenna et al. also conducted TRACE simulations with items analogous to those they used with human subjects, and found that TRACE accounted for more than 80% of the variance in the over-time fixation proportions.

**Figure 6:**
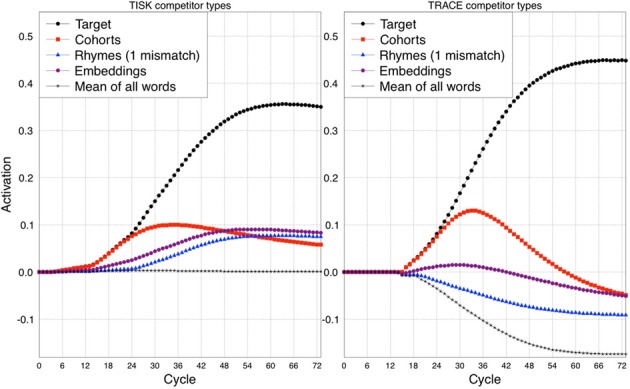
**Comparison between TISK (left panel) and TRACE (right panel) on the average time-course of activation for different competitors of a target word.** Cohort: initial phonemes shared with the target. Rhymes (1 mismatch): all phonemes except the first shared with the target. Embeddings: words that embed in the target. The average time course for all words (Mean of all words) is presented as a baseline.

In order to assess how TISK compares to TRACE in this respect, we subjected the model to simulations analogous to those used by Allopenna et al. (1998). However, rather than limiting the simulations to the small subset of the TRACE lexicon used by Allopenna et al., we actually conducted one simulation for every (correctly recognized) word in the TRACE lexicon with both TRACE and TISK. We then calculated average target activations over time, as well as the over-time average activation of all cohorts of any particular word (words overlapping in the first two phonemes), any rhymes, and words that embed in the target (e.g., for *beaker*, these would include *bee* and *beak*, whereas for *speaker*, these would be *speak*, *pea*, *peek*). Rather than selecting a single word to pair with each word as its unrelated baseline, we simply took the mean of all words (including the target and other competitors); because most words are not activated by any given input, this hovers near resting activation levels (−0.2 for TRACE, 0 for TISK). The results are shown in Figure 6.

Readers familiar with the Allopenna et al. article will notice some differences in our TRACE simulation results compared to theirs. First, we have activations below zero, while they did not. This is because Allopenna et al. followed the standard practice of treating negative activations as zero. Second, our rhyme activations remain below zero, even though they are robustly higher than those of the mean activation baseline. Having robustly positive rhyme activations in TRACE requires the use of a carrier phrase like the one used by Allopenna et al. (or a transformation to make all activations above resting level positive); without this, because there is strong bottom–up priority in TRACE, cohorts will be so strongly activated that rhyme activation will be difficult to detect. However, what really matters for our purposes is the relative activations of each competitor type, which are clearly consistent between the two models.

### 3.3. Lexical factors influencing recognition

**Figure 7:**
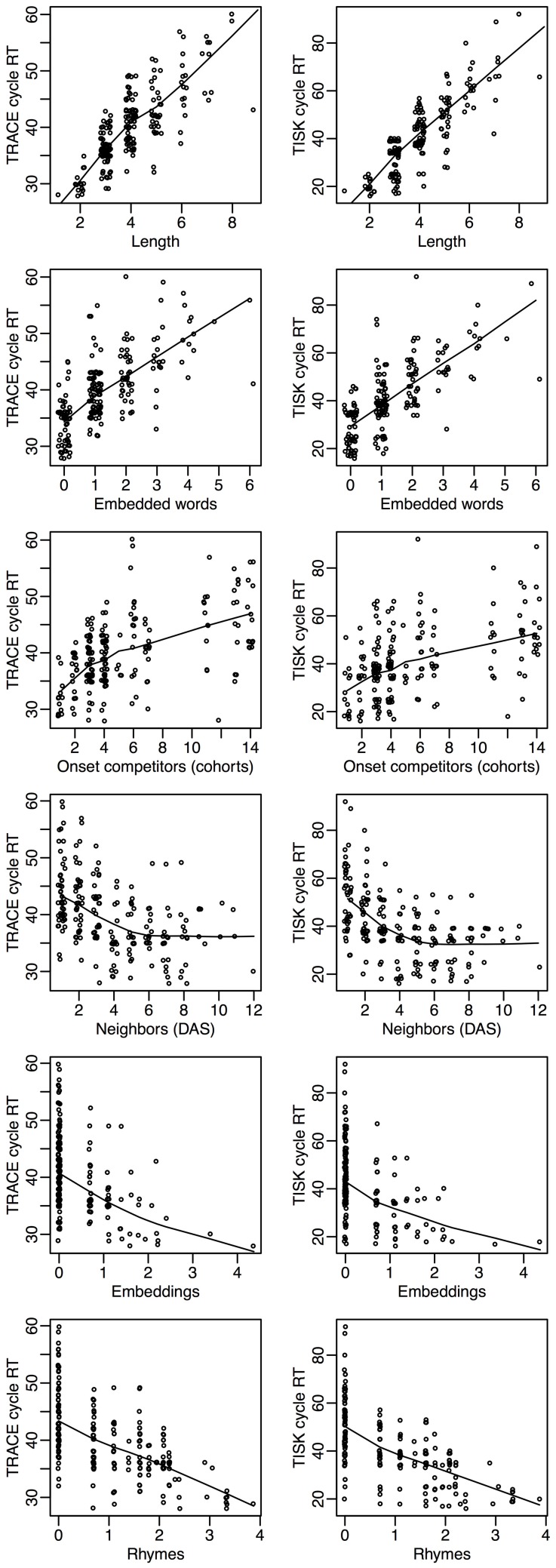
**An overview of how recognition cycles correlate with other lexical variables in TRACE (left column) and in TISK (right column).** Length: target length. Embedded words: number of words that embed in the target. Onset competitors (Cohorts): number of words that share two initial phonemes with the target. Neighbors (DAS): count of deletion/addition/subsitution neighbors of the target. Embeddings: logarithm of the number of words the target embeds in. Rhymes: logarithm of the number of words that overlap with the target with first phoneme removed.

Let's return to item level recognition times. We can probe the models more deeply by investigating how recognition times vary in each model with respect to the lexical dimensions that have attracted the most attention in the spoken word recognition literature. Figure 7 presents the correlation between recognition cycles and six standard lexical variables: the length of the target (Length), how many words it embeds in (Embeddings), how many words embed in it (Embedded), how many deletion/addition/subsitution neighbors it has (Neighbors), the number of words with which it shares 2 initial phonemes (Cohorts), and the number of words that overlap with it when its first phoneme is removed (Rhymes).

Figure 7 shows that among the six lexical dimensions considered, three are inhibitory dimensions (Length, Embedded words and Cohorts) and three are clearly facilitatory dimensions (Neighbors, Embeddings, and Rhymes). Crucially, precisely the same relationships are seen for both models, with an agreement that is not only qualitative but also quantitative.

Facilitatory variables are perhaps the most surprising, as neighborhood has long been construed as an inhibitory variable for spoken word recognition. Although the precise details are not relevant for this initial presentation of TISK, further inspection of these neighborhood effects reveals that there is an interaction of neighborhood with word length; for longer words, neighbors begin to have a facilitative effect. The crucial point is that one can see that TRACE and TISK behave in remarkably similar ways—and both make intriguing, even counter-intuitive, but testable predictions.

### 3.4. Computational resources

We will end this comparison with an assessment of the resources needed in both models. Table 1 shows the number of connections and units in TRACE and TISK, as calculated in Appendix C. The figures for TRACE are obtained by considering the number of units required per slice in the model (starting from the phoneme level, for a fair comparison with TISK which doesn't use a featural level): 14 phonemes, and, in the basic TRACE lexicon, 212 words, for 226 units. Now assuming an average of 3 phonemes per word, and allowing for connections between units at adjacent time slices, TRACE needs approximately 225,000 connections per time slice. If we make the trace 200 time slices long (which assuming 10 ms per slice would amount to 2 s, the duration of echoic memory), we need approximately 15,000 units and 45 million connections. Increasing the lexicon to a more realistic size of 20,000 words and the phoneme inventory to 40, these figures reach approximately 1.3 million units and 400 billion connections.

**Table 1:** **Estimates of the number of units and connections required in TRACE and TISK for 212 or 20,000 words, 14 or 40 phonemes, an average of four phonemes per word, and assuming 2 s of input stream**.

	**212 words 14 phonemes**		**212 words 40 phonemes**		**20,000 words 40 phonemes**	
	**TRACE**	**TISK**	**TRACE**	**TISK**	**TRACE**	**TISK**
Units	15, 067	3222	16, 800	9852	1, 336, 000	29, 640
Connections	45, 049, 733	3, 737, 313	45, 401, 600	31,718,357	>4E + 11	348, 783, 175

Next let us consider the situation in TISK. With a 2 s layer of time-specific input units (again, corresponding to the approximate limit of echoic memory), 14 phonemes and 212 words as in TRACE, TISK requires 3.2 thousand units and 3.7 million connections. This represents a 5-fold improvement over TRACE for units, and a 15-fold improvement for connections. With 20,000 words and 40 phonemes, TISK would require approximately 29,000 units (TRACE requires 45 times more) and 350 million connections (TRACE requires 1.1 thousand times more).

**Figure 8:**
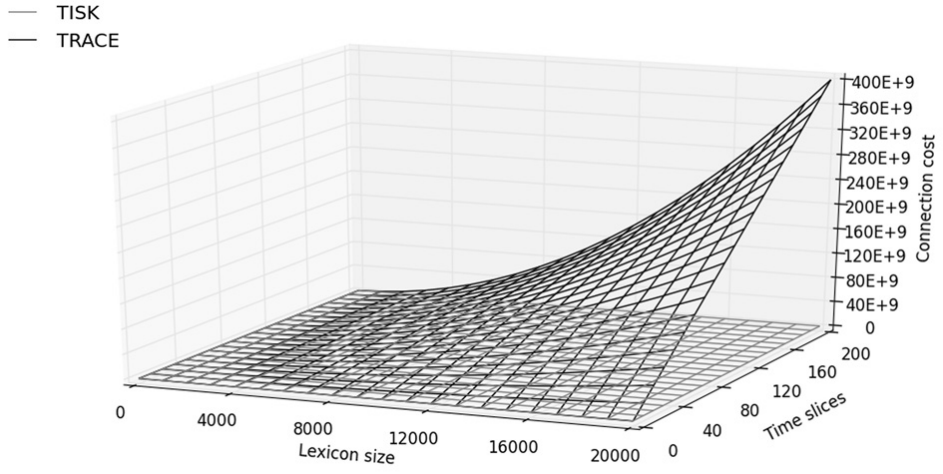
**Number of connections (*y*-axis, “connection cost”) as a function of time slices and lexical size in TISK (gray surface) and TRACE (black surface)**.

Figure 8 presents an overview of the number of connections as a function of trace duration (number of time slices) and lexicon size in TISK and in TRACE. The most striking feature already apparent in Table 1 is that TRACE shows an increase in connections which dwarfs the increase in TISK. But Figure 8 also shows that in TRACE this increase is quadratic in lexicon size and steeply linear in time slices, while connection cost in TISK looks linear in both variables with very small slopes. While Appendix B demonstrates that both functions are actually quadratic in the number of words (due to lateral inhibition at the lexical level in both models), there is still a qualitative difference in that the quadratic explosion due to the word level is not multiplied by the number of time slices in TISK, like it is in TRACE—decoupling trace duration and lexicon size was, after all, the whole point of this modeling exercise.

What is the significance of this computational economy for spoken word recognition? We would argue that it makes it easier to examine the behavior of the model at large scales. The 400 billion connections required in TRACE currently discourage any direct implementation with a realistic lexicon. However, word recognition behavior in IA models like TRACE and TISK is exquisitely sensitive to the nature of lexical competition. One should therefore not be content with effects obtained using an artificial sample of 200 words but should aim at running the model on the most realistic lexicon possible.

Depending on the precise linking assumptions one is willing to make between units and connections on the one hand, and actual neurons and synapses on the other hand (see, for instance, de Kamps and van der Velde, 2001 for a well-motivated attempt), one may or may not find that for some large but still reasonable lexicon size the connection cost in TRACE becomes larger than the sum total of all available synapses in the brain, whereas Figure 8 and Appendix B suggest that the cost in TISK would be orders of magnitude smaller and may barely make a dent in the synaptic budget.

But even leaving aside this possibility, the notion that wiring cost should come into consideration when modeling cognitive systems appears to be rather safe. Firing neurons and maintaining operational synapses has a high metabolic cost, and the pressure to perform such a ubiquitous task as spoken word recognition would seem to demand an implementation that balances cost and efficiency in the best possible way. Although the connections in TRACE or TISK are meant to be functional rather than biological, metabolic costs at the biological level constrain connectivity at the functional level: numerous functional networks as derived from human brain imaging achieve economical trade-offs between wiring cost and topological (connectivity) efficiency (Bullmore and Sporns, 2012). Recent investigations with artificial neural networks have also shown that minimizing the number of connections can improve performance by favoring the emergence of separate levels of representations (Clune et al., 2013).

## 4. Discussion

### 4.1. Spoken and visual word recognition: A bridge between orthography and phonology

In 1981, McClelland and Rumelhart presented an interactive-activation model of visual word recognition that was to be a major inspiration for the TRACE model of spoken word recognition (McClelland and Elman, 1986) and an inspiration for future generations of reading researchers. Most important is that in Figure 1 of their article, McClelland and Rumelhart sketched an overall architecture for visual and auditory word perception, describing interconnections between the two in the form of reciprocal letter-phoneme connections. This architecture clearly predicts that visual word recognition should be influenced on-line by phonological knowledge and spoken word recognition should be influenced by orthographic knowledge. Support for these predictions has since been provided by a host of empirical investigations (see Grainger and Ziegler, 2008 for a review). Strangely enough, however, attempts to implement such a bi-modal architecture have been few and far between. Research on visual word recognition has come the closest to achieving this, with the development of computational models that include phonological representations (Seidenberg and McClelland, 1989; Plaut et al., 1996; Coltheart et al., 2001; Perry et al., 2007).

With respect to spoken word recognition, however, to our knowledge no computational model includes orthographic representations, and although our TISK model of spoken word recognition is not an improvement in this respect, it was nevertheless designed with the constraint of eventually including such representations in mind. As such, TISK not only provides an answer to McClelland and Elman's question of how to avoid duplication in TRACE, but also picks up on McClelland and Rumelhart's challenge to develop a truly bimodal model of word recognition. One model has been developed along the lines initially suggested by McClelland and Elman (1986)—this is the bimodal interactive-activation model (Grainger et al., 2003; Grainger and Holcomb, 2009), recently implemented by Diependaele et al. (2010). Future extensions of this work require compatibility in the way sublexical form information is represented for print and for speech. The present work applying string kernels to spoken word recognition, along with our prior work applying string kernels to visual word recognition (Hannagan and Grainger, 2012), suggest that this particular method of representing word-centered positional information provides a promising avenue to follow. Indeed, string kernels provide a means to represent order information independently of whether the underlying dimension is spatial or temporal, hence achieving spatial invariance for visual words and temporal invariance for spoken words.

### 4.2. Testing for temporal invariance in spoken word recognition

Researchers interested in the neural representations for visual words are blessed with the Visual Word Form Area, a well-defined region in the brain that sits at the top of the ventral visual stream, and is demonstratively the locus of our ability to encode letter order in words or in legal non-words (Cohen et al., 2000; Gaillard et al., 2006) but is not selectively activated for spoken words. Until recently, the common view was that by the mere virtue of its situation in the brain, if not by its purported hierarchical architecture with increasingly large receptive fields, the VWFA was bound to achieve complete location invariance for word stimuli. However, recent fMRI studies show that, and computational modeling explains why, a significant degree of sensitivity to location is present in the VWFA (Rauschecker et al., 2012). A trained, functional model of location invariance for visual words explains why this can be so (Hannagan and Grainger, in press). In this model the conflicting requirements for location invariant and selectivity conspire with limited resources, and force the model to develop in a symmetry network with broken location symmetry on its weights (Hannagan et al., 2011). This in turn produces “semi-location invariant” distributed activity patterns, which are more sensitive to location for more confusable words (Hannagan and Grainger, in press). Thus brain studies have already been highly informative and have helped constrain our thinking on location invariance in visual words.

But attempts to proceed in the same way for the auditory modality quickly run into at least two brick walls. The first is that a clear homologue of the VWFA for spoken words has remained elusive. This might be because the speech signal varies in more dimensions than the visual signal corresponding to a visual object; a VWFA homologue for speech might need to provide invariance not just in temporal alignment, but also across variation in rate, speaker characteristics, etc. However, one study points to the left superior temporal sulcus as a good candidate for an Auditory Word Form Area (AWFA) on the grounds that this region only responded for auditory words and showed repetition suppression when the same word was spoken twice (Cohen et al., 2004), and there have been reports of invariance for temporal alignment or speaker characteristics and/or multidimensional sensitivity in the superior (Salvata et al., 2012) and medial (Chandrasekaran et al., 2011) temporal gyri. The second issue is that paradigms for testing temporal invariance are less easily designed than those which test location invariance in the visual case. Speculating from Rauschecker et al. (2012), however, we can propose a task that tests for the presence of time-specific word representations, in which subjects would be presented with a sequence of meaningless sounds where one spoken word would be embedded. By manipulating the position of this word in the sequence, one could then test whether a “blind” classifier could be trained to discriminate by their positions-in-time the different fMRI activation patterns evoked in the superior temporal sulcus. Because this decoding procedure can be applied to signals recorded from several disconnected regions of interest, this procedure would be agnostic to the existence of a well-circumscribed AWFA. TRACE and TISK both predict that the classifier should succeed with fMRI patterns evoked early on in the processing stream, i.e., at the time-specific phoneme level, but only TISK predicts that time-invariant representations should be found downstream, for lexical representations. Although the necessity for testing the existence of time-specific units is obvious in the light of the TISK model, we would argue that this has long been an urgent experimental question to ask. TRACE has been the most successful model of spoken word recognition for almost three decades now, and therefore it might be worth taking seriously the most striking hypothesis it makes of the existence of time-specific units, an hypothesis which even TISK does not succeed in completely avoiding at the phoneme level.

### 4.3. Previous models and alternative approaches to temporal order

We claimed previously that TRACE has the greatest breadth and depth of any extant model of spoken word recognition. Of course, there are models whose proponents argue that they have solved key problems in spoken word recognition without using TRACE's inefficient time-specific reduplication strategy. We will review a few key examples, and consider how they compare with TRACE and TISK.

Norris (1994), Norris et al. (2000), and Norris and McQueen (2008) introduced Shortlist, Merge, and Shortlist B, the first two being IA network models and the latter a Bayesian model of spoken word recognition. All three models share basic assumptions, and we refer to them collectively as “the Shortlist models.” Contrary to TRACE, the Shortlist models are entirely feedforward. They also make a critical distinction between words and tokens, the latter being time-specific entities that instantiate the former, time-invariant lexical templates. The reduplication of the lexical level that afflicts TRACE is avoided in these models by assuming that only a “short list” of tokens is created and wired on-the-fly into a “lattice” of lexical hypotheses. These models have a sizable lexicon (even a realistic 20,000 word lexicon in the case of Shortlist B), and although they have not been applied to the full range of phenomena that TRACE has, they have successfully simulated phenomena such as frequency and neighborhood effects. Unfortunately, because no computational mechanism is described that would explain how the on-the-fly generation and wiring of tokens could be achieved, the account of spoken word recognition provided by Shortlist is still essentially promissory.

Other approaches to temporal order use fundamentally different solutions than TRACE's reduplication of time-specific units. Elman's (1990) simple recurrent network (SRN) may be foremost among these in the reader's mind. The SRN adds a simple innovation to a standard feedforward, backpropagation-trained two-layer network: a set of context units that provide an exact copy of the hidden units at time step *t*-1 as part of the input at time *t*, with fully connected, trainable weights from context to hidden units. This feedback mechanism allows the network to learn to retain (partial) information about its own state at preceding time steps, and provides a powerful means for sequence learning. However, while SRNs have been applied to speech perception and spoken word recognition (most notably in the Distributed Cohort Model: Gaskell and Marslen-Wilson, 1997, but for other examples see Norris 1990, and Magnuson et al. 2000, 2003), so far as we are aware, no one has investigated whether SRNs can account for the depth and breadth of phenomena that TRACE does (though SRNs provide a possible developmental mechanism since they are learning models, and the Distributed Cohort Model has been applied to semantic phenomena beyond the scope of TRACE).

Another approach is the cARTWORD model of Grossberg and Kazerounian (2011), where activity gradients specific to particular sequences can differentiate orderings of the same elements (e.g., ABC vs. ACB, BAC, etc.). However, this mechanism cannot represent sequences with repeated elements (for example, it cannot distinguish ABCB from ABC, as the second B would simply provide further support for B rather than a second B event), which makes it incapable of representing nearly one third of English lemmas. Furthermore, it is premature to compare this approach to models like TRACE, since it has been applied to a single phenomenon (phoneme restoration) with just a few abstract input nodes and just a few lexical items; thus, we simply do not know whether it would scale to handle realistic acoustic-phonetic representations and large lexicons, let alone the broad set of phenomena TRACE accounts for (see Magnuson submitted, for detailed arguments and simulations showing that the supposed failures of TRACE to account for phoneme restoration phenomena reported by Grossberg and Kazerounian 2011, were the result of flawed simulations, not a problem with TRACE). Note that a similar activity gradient approach in visual word recognition (Davis, 2010) has also been attempted, with similar limitations.

### 4.4. The utility of interactive activation models

Because spoken word recognition is a slowly acquired skill in humans, any model of it should eventually strive to incorporate some kind of learning algorithm that explains how the representations necessary to solve the task have matured. Unlike SRNs though, models such as TRACE and TISK do not comply to this requirement. On the other hand and until proven the contrary TRACE vastly outperforms SRNs in explanatory power while having the advantage of being more transparent. We would argue that IA models and learning models like SRNs should be construed as complementary approaches to spoken word recognition. Imagine SRNs were demonstrated to account for similar depth and breadth as TRACE. We would still be left with the puzzle of how they do so. Unpacking the complex composites of cooperative and competitive wiring patterns that would develop would be no mean feat. This is where we find interactive activation models like TRACE and TISK especially useful. The IA framework allows one to construct models with levels of organization (the representational levels) with inter- and intralevel interaction governed by discrete parameters. This allows one to generate hypotheses about which aspects of the model are crucial for understanding some phenomenon (e.g., by investigating which model parameters most strongly generate a key behavior), or about which level of organization may be perturbed in a particular language disorder (Perry et al., 2010; Magnuson et al., 2011). One modeling approach that is likely to be productive is to use simpler frameworks like IA models to generate hypotheses about key model components in some behavior or disorder, and then to seek ways that such behaviors or disruptions might emerge in a more complex model, such as an SRN or another type of attractor network (cf. Magnuson et al., 2012). Similarly, TISK provides a testbed for investigating whether a string kernel scheme is a robust basis for spoken word recognition. For example, the success of string kernel representations in TISK might suggest that we should investigate whether the complex wiring SRNs learn approximates string kernels.

### 4.5. Relationship between trace and tisk

One might be surprised that TISK and TRACE display such similar behavior despite the lack of feedback in the former and its presence in the latter. Feedback in models of spoken word recognition is a controversial topic (McClelland et al., 2006; McQueen et al., 2006; Mirman et al., 2006a), which we do not address here; our aim is to see whether a model with a radically simpler computational architecture compared to TRACE can (begin to) account for a similar range of phenomena in spoken word recognition. However, this resemblance despite feedback is less surprising than it may seem. Indeed, it has been known for several years that the feedback contribution to word recognition in TRACE is limited given noise-free input (Frauenfelder and Peeters, 1998): simulations show that feedback makes the model more efficient and robust against noise (Magnuson et al., 2005). It also provides an implicit sensitivity to phonotactics—the more often a phoneme or n-phone occurs in lexical items, the more feedback it potentially receives—and it is the mechanism by which top–down lexical effects on phoneme decisions are explained in TRACE. None of these effects were considered in this article, which focused on core word recognition abilities and lexical competition effects. We acknowledge that without feedback, TISK will not be able to simulate many top–down phenomena readily simulated in TRACE. Future research with TISK will explore the impact of feedback connections.

### 4.6. Limitations and next steps

The aim of this study was to improve on one particularly expensive aspect of the TRACE model without drastically affecting its lexical dynamics, or diminishing its explanatory power. We have demonstrated that a radically different approach to sequence representation, based on string kernels, provides a plausible basis for modeling spoken word recognition. However, our current model has several obvious limitations.

First, to apply TISK to the full range of phenomena to which TRACE has been applied will require changes, for example, in the input representations for TISK. As we mentioned above, we used single-point inputs for TISK rather than the on- and off-ramping, over-time inputs in TRACE that also give the model a coarse analog to coarticulation. An input at least this grain will be required to apply TISK to, for example, subcategorical mismatch experiments that TRACE accounts for (Dahan et al., 2001b).

Second, TISK's levels and representations are stipulated rather than emergent. Our next step will be to examine whether codes resembling string kernels emerge when intra-level weights are learned rather than stipulated. What learning algorithm could find the set of weight values under which TISK and TRACE have been shown to achieve close to perfect recognition? Is there more than one such set, and do they make different predictions from the existing fine-tuned solutions? There are a few results that suggest the way forward. For instance, there are demonstrations that Hebbian learning applied at the lexical level in TRACE can help explain short term phenomena in spoken word recognition (Mirman et al., 2006b). If Hebbian learning is indeed active on short scales, there are no reasons to doubt that it will be involved on longer time-scales, slowly shaping the landscape of inhibition between words, which forms the basis for much of the behaviors explored in this article.

Third, a problem shared by all models of word recognition is that it is not clear how to scale from a model of word recognition to higher levels, e.g., to a model of sentence comprehension. Because TISK's word level is time-invariant, there is no obvious way to generate ngrams at the word level. However, TISK and TRACE, like other models capable of activating a series of words over time given unparsed input (i.e., word sequences without word boundary markers) should be linkable to parsing approaches like “supertagging” (Bangalore and Joshi, 1999; Kim et al., 2002) or the self-organizing parser (SOPARSE) approach of Tabor et al. (e.g., Tabor and Hutchins, 2004). Note that a common intuition is that SRNs provide a natural way of handling sequential inputs from acoustics to phonemes to words. However, it is not clear that this translates into a comprehensive model of the entire speech chain. It is not apparent that you could have a single recurrent network that takes in acoustics and somehow achieves syntactic parsing (let alone message understanding) while producing human-like behavior at phonetic, phonological, lexical levels. These are non-trivial and unsolved problems, and despite the intuitive appeal of recurrent networks, remain unanswered by any extant model.

Finally, it is notable that we have not implemented feedback yet in TISK. This renders TISK incapable of accounting for top–down lexical effects on phoneme decisions. However, as Frauenfelder and Peeters (1998) and Magnuson et al. (2005) have demonstrated, feedback plays little role in recognition given clear inputs. When noise is added to a model like TRACE, feedback preserves speed and accuracy dramatically compared to a model without feedback. While feedback also provides a mechanistic basis for understanding top–down effects, it is also remarkable that at least one effect attributed to feedback in TRACE (rhyme effects; Allopenna et al., 1998) emerges in TISK without feedback. This suggests that in fact examining which, if any (other), putatively top–down effects emerge without feedback in TISK will be a useful enterprize. Given, however, the remarkable fidelity to TRACE that TISK demonstrates over a broad swath of phenomena, it is clear that feedback need not be included in this first assay with TISK.

## 5. Conclusion

Twenty-seven years after Elman and McClelland introduced the TRACE model, we have endeavored to answer the question of how to dispense with time-duplication, and have presented an alternative that preserves TRACE-like performance on spoken word recognition while using orders of magnitude less computational resources. Perhaps more importantly, the particular structures and mechanisms that achieve time-invariance in TISK construct new and intriguing bridges between visual and spoken word recognition.

## Funding

Thomas Hannagan and Jonathan Grainger were supported by ERC research grant 230313.

### Conflict of interest statement

The authors declare that the research was conducted in the absence of any commercial or financial relationships that could be construed as a potential conflict of interest.

## Appendix

### A. Parameters of the model

**Table:**

**Name**	**Value**	**Description**
Times	10	Number of time-specific slots (for input and time specific phonemes)
Istep	10	Pace of input stream (a new input is introduced every “istep” cycles)
Deadline	100	Deadline
DecayP	0.01	Decay rate for time-specific phonemes
DecayNP	0.01	Decay rate for time-invariant nphones
DecayW	0.05	Decay rate for time-invariant words
Gap	max	Authorized gap between phonemes in time-invariant nphones
		(e.g., if gap = 1, “/bark/” = “/ba/,” “/ar/,” “/rk/”;
		if gap = 2, “/bark/”= ‘/ba/,” “/br/,” “/ar/,” “/ar/,” “/ak/,” “/rk/”).
PtoNPexc	0.1	Time-specific phoneme to time-invariant nphone excitation
PtoNPthr	6	Time-invariant nphone activation threshold
NPtoNPinh	0	Lateral inhibition between nphones
NPtoWexc	0.05	Excitation from time-invariant nphone (“/ba/”) to words (“/bark/”)
NPtoWscale	Wordlength	Scaling factor for NPtoW connections (here, set to word length)
WtoNPexc	0	Excitation from words (“/bark/”) to time-invariant nphone (“/ba/”)
1PtoWexc	0.01	Excitation from 1-phone (“/a/”) to words (“/bark/”)
Wto1PExc	0	Excitation from words (“/bark/”) to 1-phone (“/a/”)
WtoWinh	−0.005	Lateral inhibition between words

### B. Sizing trace

Recall that TRACE duplicates each feature, phoneme, and word unit at multiple time slices. Features repeat every slice, while phonemes and words repeat every three slices. Figure 2 illustrates reduplication and temporal extent of each unit type. For completeness we will include the feature level in our sizing of TRACE, although it will not be taken into account in our comparison with TISK. In the following, *S*, *F*, *P*, and *W* will, respectively stand for the number of time slices, features, phonemes and words in the model.

#### B.1. Counting units

Because there is a bank of *F* features aligned with every slice, there are *SF* feature units. For phonemes, given that we have *P* time-specific units every three slices, for a total of *P*(*S*/3). For words, we have *W* time-specific units every three slices, for a total of *W*(*S*/3).

The total number of units as a function of *S*, *F*, *P*, and *W* can therefore be written: *SF* + *P*(*S*/3) + *W*(*S*/3) = *S*(*F* + *P*/3 + *W*/3) We see that the cost in units is linear in all of these variables, and that for 201 time slices, 212 words, 14 phonemes, and 64 feature units the TRACE model requires 12,633 + 938 + 14,204 = 27,805 units.

#### B.2. Counting connections

We start by counting the feature-phoneme connections. There are seven features per phoneme on average (vowels, fricatives and liquids don't use the burst parameter, but some phones take two values within a feature level). Let us count how many phoneme units overlap with each slice. From Figure 2, we can see that two copies of each phoneme overlap with each time slice. Therefore, there are seven (features) ^*^2 (copies) ^*^*P* feature-phoneme connections per slice, which results in 14 PS feature-phoneme connections in the model.

Let us proceed to phoneme-word and word-phoneme connections. Words are four phonemes long on average, and there are *W*(*S*/3) word units. But each of those units receives input not just from the four phonemes that are maximally aligned with it, but also the phonemes to the left and right of the maximally aligned phonemes. Thus, the total number of phoneme-word connections will be 3(*S*/3)*Wp* = *SWp*, where *p* is the number of phonemes per word. There will be an equal number of feedback connections from words to phonemes, for a total count of 4*SW* Phoneme-phoneme connections.

Next we consider the phoneme–phoneme connections. Each phoneme unit has an inhibitory link to each phoneme unit with which it overlaps. We can see from Figure 2 that three copies of each phoneme overlap any given slice. So for each phoneme unit aligned at a given slice, it will have 3*P* − 1 outgoing inhibitory links (we subtract 1 for the unit itself). We do not need to count incoming connections; these are included when we multiply by the number of phoneme units. This results in a total count of *PS*(*P* − 1/3) word–word connections.

Just like phonemes, each word unit has an inhibitory link to each word unit with which it overlaps. The number of copies of any word that will overlap with a given slice will vary with word length, as can be seen in Figure 2. We can also see from Figure 2 that words span six slices per phoneme. Recall that words are duplicated every three slices. For the 2- and 3-phoneme long examples in Figure 2, we can determine that the number of copies of each word of length *p* that overlap with a given third slice (that is, an alignment slice, or a slice where one copy of the word is actually aligned) would be 1 + 2(2*p* − 1) (the first 1 is for the unit aligned at the slice), i.e., 4*p* − 1. So a word unit at an alignment slice will have (4*p* − 1)*W* − 1 outgoing inhibitory connections. Therefore we arrive at a count of *W*(*S*/3)(4*p* − 1)*W* − 1) word–word connections, which for an average word length of four phonemes amounts to *SW*(5*W* − 1/3). All in all, we arrive at the following formula for the total connection count in TRACE: Total = 14PS + 4*SW* + *PS*(*P* − 1/3) + *SW*(5*W* − 1/3) = *S*(14*P* + *W* + *P*(*P* − 1/3) + *W*(5*W* − 1/3)) = *S*(*P*(*P* + 41/3) + *W*(5*W* + 2/3)) = *S*[(*P*^2^ + 41/3*P*) + (5*W*^2^ + 2/3*W*)].

(3) $$\begin{array}{l} c_{\text{TRACE}}=14PS+4SW+PS(P-1/3)+SW(5W-1/3)\\ \;=S(14P+W+P(P-1/3)+W(5W-1/3))\\ \;=S(P(P+41/3)+W(5W+2/3))\\ \;=S[(P^{2}+41/3P)+(5W^{2}+2/3W)] \end{array}$$

According to our calculations, the cost in connections is therefore a quadratic function of *P* and *W* (due to lateral inhibition at the phoneme and word levels), and a linear function of *S* (due to limited overlap of units over time slices). In particular, with the standard parameters of 212 words, 14 phonemes, a mean word length of 4 phonemes, and 67 alignment units the TRACE model requires 45,573,266 connections.

### C. Sizing tisk

TISK has three levels: a time specific phoneme level, a time-invariant string kernel level (tisk, after which the model is named), and a time-invariant word level. TISK doesn't have a feature level, and instead the output of such a level is emulated by a wave of net inputs that arrives to the time-specific phoneme level at a regular pace. A feedforward symmetry network operates the transition between the time-specific phoneme level and the nphone level. There are positive feedforward and feedback connections between the nphone and word levels, and lateral inhibitory connections within them, although in practice only the word level has non-zero inhibitory connections and they are restricted to neighbors. The cuts in computational resources are mostly due to the symmetry network, and to a lesser extent, to the limited use of lateral inhibition.

#### C.1. TISK units

Because only one level is time-specific in TISK, the notion of alignment doesn't have course anymore. Therefore the number of time-specific phonemes is simply given by the number of phonemes multiplied by the number of time slices, or *PS*. With 14 phonemes and, 201 slices, this amounts to 2814 time-specific phoneme units. The nphone level hosts time-invariant phonemes and all possible diphones (even phonotactically illegal ones), and therefore uses *P* + *P*^2^ units, which for *P* = 14 means 210 units. Finally the word level counts *W* units, one for each word in the lexicon, and *W* is set to 212 throughout most simulations. The total number of units in the model is therefore *PS* + *P* + *P*^2^ + *W* = *P*(*P* + *S* + 1) + *W* = 3236 units. *W* time-invariant word units (212). *P* + *P*^2^ time-invariant n-phone units (*P* 1-phones and *P*^2^ diphones; = 210). Total units at basic parameters: 1360.

#### C.2. TISK connections

We only count non-zero connections throughout. We start by sizing connections in the symmetry network (Figure 3). A time-specific phoneme unit sends a connection to an nphone unit if and only if it is a constituent of this unit (for instance, *A*_2_ sends a connection to A, AB, BA, and AA, but not to B). There are 2*P* − 1 diphones that start or end with a given phoneme, and one time-invariant phoneme, so a given phoneme at time t will send 2*P* − 1 + 1 = 2*P* connections, and multiplying this by the number of time specific phonemes *PS*, we see that the total number of connections is 2*P*^2^*S*. From this, however, we must remove all zero connections: unit *A*_1_ (resp. *A*_*T*_) should not give evidence for diphone units that end with A (resp. that start with A), and therefore gradient coding assigns zero to these connections. We see that these cases only occur at the first and last time slices (implying that there are more than two time slices), and that for a given phoneme, *P* − 1 connections are concerned, resulting in 2*P*(*P* − 1) zero connections. There are therefore 2*P*^2^*S* − 2*P*(*P* − 1), or 2*P*(*SP* − *P* + 1), phoneme-to-nphone connections in the symmetry network (with 14 phonemes and 201 time slices, this amounts to 78,428 connections).

We must now count the number of gating connections in the symmetry network. To prevent spurious activations at the nphone level, the symmetry network uses gating connections. These are hard-wired connections that originate from time specific phonemes, and inhibit some connections between time-specific phonemes and time-invariant nphones. Specifically, a given phoneme at a given time slice will inhibit all connections at later time slices that originate from the same phoneme and arrive to a diphone that begins with that phoneme (and does not repeat). Because there are *P* − 1 diphones that start with a given phoneme and do not repeat, and there are *P* phonemes at a given time slice, *P*(*P* − 1) connections must be gated at any time slice after the one considered, or for *S* > 2:

(4) $$\begin{array}{l} c_{\text{gating}}=P(P-1)(S-1)+P(P-1)(S-2)+\ldots +\;P(P-1)(1)\\ \;=P(P-1)\sum_{s=1}^{S-1}s\\ \;=\frac{P(P-1)S(S-1)}{2} \end{array}$$

With 14 phonemes and 201 time slices, this amounts to 3,658,200 gating units. The total in the time specific part of the network is therefore of 3,658,200 + 78,428 = 3,736,628 connections (Note that the formulas obtained here were verified empirically by direct inspection of the number of connections in the model for various number of time slices, and were found to be exact in all cases). We now proceed to count connections in the time invariant part of the network, first noticing that because lateral inhibition at the nphone level was set to zero, we only need to count the connections between the nphone and the word level, as well as the lateral connections within the word level. However, in TISK these numbers will depend not only on the size of the lexicon and the number of nphones, but critically also on the distribution of nphones in the particular lexicon being used, so that we are reduced to statistical approximations. Empirically, we find that an average word connects to 9.5 nphones in TISK, leading to an estimate of 9.5 W feedforward connections between the nphone and word level. Similarly, simulations show that the number of lateral inhibitory connections at the word level in TISK is 0.8*W*(*W* − 1). Therefore the number of connections in the time-invariant part of the model reaches 0.8*W*^2^ − 0.8*W* + 9.6*W* = 0.8*W*^2^ + 8.8*W*. With a lexicon of 212 words, this amounts to 37,800 connections.

All in all, we arrive at the following expression for the number of connections in TISK for *S* > 2:

(5) $$c_{\text{TISK}}=2P^{2}S-2P(P-1)+\frac{P(P-1)S(S-1)}{2}+W(0.8W+8.8)$$

which amounts to 3,774,428 connections using our usual assumptions on *S*, *P*, and *W*. It can be seen when this expression is developed that it is quadratic in *S*, *P*, and *W*. This would seem to be a setback compared to the expression obtained for TRACE, which is only quadratic in *P* and *W* but linear in *S*. However, *S* is *orders* of magnitudes smaller than *W*, and what we obtain in exchange of this quadratic dependence to *S* is to decouple the *S* and *W* factors, reflecting the fact that in TISK the lexicon is not duplicated for every time slice anymore. Consequently there is a substantial gain in connections when switching from TRACE (45,573,266) to TISK (3,774,105) connections, the latter having ten times less connections, a gain of one order of magnitude which improves with lexicon size to reach an asymptota at three orders of magnitude.
